# The Week's Work

**Published:** 1910-02-12

**Authors:** 


### The Week's Work.

### A DANIEL OF TO-DAY.

In the course of his paper, read at Kensington, on "The Administration of a Voluntary Hospital: Its Use, Misuse, and Abuse," published in the *British Medical Journal* of the 5th inst., Mr. E. W. Morris, Secretary of the London Hospital, begged his audience of their charity "to deal with him as kindly as did the other lions with the Daniel of long ago." Our modern Daniel has written a most interesting paper, which summarises many of the principles which are generally accepted by those who know most about hospital management in this country. He further formulates some new points, some of which we do not think calculated to prove generally acceptable. The brief sketch which he gives of the administration of the London Hospital is well done, but it omits to show that the honorary medical staff there take no practical part in the management of the hospital and that they are excluded from membership of its committees. Yet this exclusion of the medical element from the working committees is a special feature of the London Hospital, which makes that institution almost unique among British voluntary hospitals. If Mr. Morris had had experienee of the working of several voluntary hospitals where a different system prevails, some of the views he expresses would no doubt have been modified. We are glad to notice that he strongly enforces the need for the introduction of the reforms which we have advocated in dealing with certain hospital problems which press. The London Hospital has power under its special Act to admit paying patients, and though its situation makes the successful adoption of this system unusually difficult, Mr. Morris is convinced that the existing system of voluntary hospital management should be modified in this direction. He says that all hospitals, whether voluntary, municipal, or State, should be placed under a Health Department, which was the late Dr. B. Richardson's great idea, and he never fails to urge on public attention the importance of establishing a Minister of Health. Mr. Morris's paper is one which should be read. It cannot fail to prove of general interest, and to be welcomed by members of the medical profession and hospital managers.

### FOUR CHANGING FORCES.

Mr. Morris insists that the four main forces of the voluntary hospitals are the sick, the subscribers, the honorary staff, and the students, each of which differs widely from what it was half a century ago. The sick poor, who formerly represented those who were without means, and who had no place of shelter when ill except the hospitals, have ceased for the most part to enter voluntary hospitals, being at present accommodated in the Poor Law infirmary. The present patients of the voluntary hospitals are drawn largely from the wage-earning classes and small shopkeepers, who for the most part maintain that, as they get free education for their children, they are entitled also to receive free medical relief. This change of opinion in the classes referred to, from the old standard of self-respecting thrift to one of socialistic dependence upon charity or State or rate support, is an evil for which the voluntary hospitals are largely responsible, and they ought, therefore, to make a supreme effort to restore the moral fibre of the people by reforming root and branch their present system of medical relief. The subscribers who formerly gave with unquestionable pleasure to a voluntary hospital are beginning to realise the dangers and abuses attaching to this change of opinion on the part of the classes from which the patients are mainly drawn, and there are signs that an increasing number of them are awakening to the fact that either a reform of the present system of relief must speedily be introduced or they will not be justified in continuing to give to the voluntary hospitals in the future, as in the past, with ever-increasing generosity. The honorary medical staff, who are paid honorariums for their services at the Royal, and a few other hospitals, like the Brompton, are regarded nowadays by the patients and the public as very fortunate gentlemen indeed. The public now realises "that it pays a medical man a hundredfold to be on the staff of a hospital, and from a former state of adoration for the man who gave his services to the sick and suffering they have gone to the other extreme and fancy that every hospital doctor is very much on the make." Naturally Mr. Morris has something interesting to say which exalts the position of the hospital dispenser, and he insists that the duties and responsibilities of this officer have increased enormously. Mr. Morris does not agree, of course, with the crude change in public opinion in regard to free medical service by the honorary staff, though he does not consider it necessary that they should be paid for some years to come at any rate. We fear that unless the payment of an honorarium to the honorary medical staff is adopted by all the principal voluntary hospitals, the changes so urgently called for in the present system of medical relief may be indefinitely delayed. We are glad that Mr. Morris should emphasise the importance to the patient and to the whole administration of the presence of medical students in a hospital. These pertinacious critics, who want to know all about causes, act as the fierce light which beats on all the surgeon or physician does, thus constituting them a power for good. So the patients gain in every way by the presence of the student. It is a little remarkable that Mr. Morris should, however, suggest the exclusion of the students from the wards in which pay patients are received. Why?

### SOME PRACTICAL SUGGESTIONS.

Mr. Morris practically recommends that free medical relief in hospitals should be confined to the class of cases we defined in our articles published in our issues of January 22 and February 5, pp. 488 and 546 respectively. He rightly insists upon the co-operation of all hospital agencies one with the other; but to effect this object we infinitely prefer the plan, recommended in the articles just referred to, to the establishment of a State department with relieving officers or almoners to whom any person needing hospital care should first apply. Such a system would never work. The voluntary system, as Mr. Morris admits, to endure must maintain its individuality. The Minister of Health might properly have inspection jurisdiction over all hospitals, and it should be possible with the aid of such a department to make arrangements whereby any member of the public needing hospital care could ascertain at once where there was a vacant bed into which he could be received. This important detail ought to be attended to at once by the Central Hospital Council, which could readily render this service to the inhabitants of the Metropolis, and so bring London into line with every great Continental city in this respect. Mr. Morris thinks State aid will come, but not State control. Representation, however, always has followed, and always will follow, monetary aid for the maintenance of any object. The only State aid, apart from State control, which the voluntary hospitals may expect is by way of grants to enable them to defray the cost of erecting paying wards upon a business basis; that is, one which provides for the repayment of the grant on a sinking fund basis out of the patients' payments. We agree with Mr. Morris that the out-patient departments of all hospitals must decrease, and, as we hope, ultimately disappear. As they exist to-day they are a grave abuse and a grave injustice, being morally wrong in principle and absolutely unsatisfactory from the patients', the profession's, and the public's point of view. We have not space to do more than indicate a few of the points covered by Mr. Morris's interesting paper. Some of them, especially those relating to the aspects of the voluntary system, we have examined and disposed of in recent issues. Mr. Morris's paper entitles him to the warm congratulations of all who take an interest in the practical reform of the present unsatisfactory system of British medical relief.

### HOSPITALS AND THE PUBLIC.

A thoughtful and interesting paper was recently read by Dr. Willis Ford before an American medical society upon the advantages of a small hospital. Dr. Ford has had forty years' experience of hospital work, and his reminiscences and reflections are naturally worth listening to. Comparing the hospital of to-day with the institutions existing in the days when he was a house physician, he finds that the greatest change has undoubtedly taken place in the nursing department. That change is as apparent in America as it is here, and is responsible very largely for the "change in the opinions of the public regarding the desirability of hospital care." In the old times removal to a hospital was dreaded; nowadays there is such a thing as hospital abuse, though it is worth while to remember that even in the Middle Ages hospital abuse was complained of. "The fact that it has been recognised that a large amount of money must be expended to maintain a hospital that pretends to anything like scientific work in these days," remarks Dr. Ford, "and that instruments of precision and appliances used in the care of sick people and apparatus for mechanical therapeutics must be supplied in order to make a hospital a success in any way, has furnished an argument which physicians have used, that many cases of sickness can be better cared for in hospitals than in private houses. The people, therefore, who now go to hospitals are very different from those whom we used to see even in the best of small private hospitals fifteen years ago, so that it has come about at last that hospitals agree to furnish such attention, appliances, and skilled care as but few households can secure, even if there is plenty of money to expend. This has naturally fostered the tendency to the development of small hospitals.

### THE COTTAGE HOSPITAL.

In this growth of small hospitals, and especially their construction in small towns, there are some very important facts which Dr. Ford thinks the profession ought to recognise. "Among the laity," he writes, "it is now a common expression that if you go to a hospital you are sure to receive better care than you can in your own house; and yet it is perfectly apparent to the physicians who are working in hospitals that this depends entirely upon whether the hospital is thoroughly equipped, and whether it has funds enough to supply wholesome and even generous diet, and whether it is in the control of physicians who have skill and experience above the average man in their profession. There is no question but that the influence of the small hospital upon the community is a wholesome influence." He points out that cottage hospitals are relatively expensive, and that their expense is likely to increase in the near future if they are really to remain up to date. "Without ample endowment the small hospital cannot act the rôle of benefactor. Forced economy usually means doing without the proper food for the sick and failure of proper surgical technique." In the circumstances it should always be very seriously considered "whether a small gift, the result of a transient enthusiasm or an evanescent charitable impulse, should be accepted to start a small hospital without adequate provision for its maintenance."

### THE DRUG MARKET

Few changes of importance in the prices of drugs and chemicals have taken place during the week, and business has been quieter than was expected. There is a probability that the value of opium will recede, and laudanum and other preparations of this drug should in the meantime be purchased in small quantities only. There is, however, some uncertainty about the market, as everything depends on the weather in the producing districts during the next few weeks; nevertheless, it does not seem very probable that prices will advance. The price of morphine is slightly lower. The price of quicksilver has been reduced, and this may be followed by a slight reduction in the prices of calomel, corrosive sublimate, and other salts of mercury, but not necessarily. Ipecacuanha continues very dear, and quotations for the liquid extract and other preparations have been advanced.

